# Donor and Procedural Factors Associated With Citrate Toxicity During Plateletpheresis

**DOI:** 10.7759/cureus.117188

**Published:** 2026-09-29

**Authors:** Abrar Kabir Shishir, A B M Al-Mamun, Ummea Sayeba Hassan, Sadiya Nahar Shorna, M M Zahidul Islam, Md Zubayer Ferdous, Ferdousy Binte Ali Borna, Md. Faizul Ahasan, Kashfia Islam, Shanaz Karim

**Affiliations:** 1 Department of Transfusion Medicine, Ahsania Mission Cancer and General Hospital, Dhaka, BGD; 2 Department of Transfusion Medicine, Dhaka Medical College Hospital, Dhaka, BGD; 3 Department of Transfusion Medicine, Bangladesh Institute of Health Sciences General Hospital, Dhaka, BGD; 4 Department of Transfusion Medicine, Meherpur 250 Bedded General Hospital, Meherpur, BGD; 5 Department of Transfusion Medicine, Rangpur Medical College Hospital, Rangpur, BGD; 6 Department of ENT and Head-Neck Surgery, Cumilla Medical College Hospital, Cumilla, BGD; 7 Department of Pharmacology and Therapeutics, Ibrahim Medical College and Bangladesh Institute of Research and Rehabilitation in Diabetes, Endocrine and Metabolic Disorders (BIRDEM) General Hospital, Dhaka, BGD

**Keywords:** body mass index, calcium carbonate, citrate toxicity, donor safety, plateletpheresis

## Abstract

Background

Citrate anticoagulation during plateletpheresis can reduce ionized calcium and cause neuromuscular or gastrointestinal symptoms. Identifying donor and procedure-related factors may support earlier recognition and prevention. This study assessed factors associated with clinically identified citrate toxicity among plateletpheresis donors.

Methods

This analytical observational cross-sectional study included 60 plateletpheresis donors at Dhaka Medical College and Hospital, Bangladesh. Citrate toxicity was defined by any prespecified symptom or sign during the donation encounter. Crude and adjusted prevalence ratios (PRs) were estimated using modified Poisson regression with robust variance. The parsimonious adjusted model included pre-procedure oral calcium exposure, body mass index (BMI), pre-donation platelet count, and anticoagulant citrate dextrose solution A (ACD-A) volume. Calcium administration was non-randomized, and total and ionized calcium were not measured.

Results

All 60 donors were male; 28 (46.7%) developed citrate toxicity. Compared with donors without toxicity, affected donors had lower mean BMI (24.03 versus 25.56 kg/m^2^; p=0.020), lower pre-donation platelet count (2.53 versus 3.23×10^11^/L; p<0.001), and greater mean ACD-A exposure (333.39 versus 259.13 mL; p<0.001). In adjusted analysis, absence of pre-procedure calcium was associated with higher toxicity prevalence (adjusted PR {aPR} 4.42, 95% CI: 2.59-7.56), each 1 kg/m^2^ higher BMI with lower prevalence (aPR: 0.91, 95% CI: 0.85-0.98), and each 50 mL higher ACD-A volume with higher prevalence (aPR: 1.65, 95% CI: 1.24-2.18). Pre-donation platelet count was not independently associated after adjustment (aPR: 0.79 per 0.5×10^11^/L, 95% CI: 0.56-1.12).

Conclusions

Clinical citrate toxicity was common and was associated with absence of pre-procedure calcium, lower BMI, and greater ACD-A volume. These findings identify potential monitoring markers but do not establish causality because procedural factors and symptoms were recorded within the same encounter and ionized calcium was not measured. The calcium association should be interpreted as exploratory and hypothesis-generating, rather than as evidence of prophylactic efficacy.

## Introduction

Platelet transfusion is an essential component of supportive care for patients with hematologic malignancies, chemotherapy-associated thrombocytopenia, marrow failure, major bleeding, and platelet dysfunction. Plateletpheresis collects a therapeutic platelet dose from a single donor and may reduce recipients' exposure to multiple donors compared with pooled whole-blood-derived platelets [1]. Demand for platelet components has increased with the expansion of intensive oncology and transplant care, reinforcing the need to maintain a safe and willing donor population [2,3].

Citrate, most commonly administered as anticoagulant citrate dextrose solution A (ACD-A), prevents clotting in the extracorporeal circuit by chelating ionized calcium. The resulting transient reduction in biologically active calcium can alter neuromuscular and cardiac membrane excitability. Clinical manifestations range from circumoral or acral paresthesia and nausea to muscle cramping, carpopedal spasm, tetany, seizures, and arrhythmia in severe cases [4]. Serial biochemical studies in plateletpheresis donors demonstrate that ionized calcium typically falls during the procedure, although the magnitude, recovery, and clinical expression vary among donors and devices [5-7].

Most apheresis adverse events are mild, but uncomfortable reactions may discourage repeat donation. Reported risk factors include lower body size, first-time donation, larger processed blood volume, longer procedure duration, and greater citrate exposure [8-10]. In a retrospective study from Bangladesh, donor-related and procedure-related adverse events were documented during plateletpheresis, including perioral tingling, underscoring the local relevance of donor vigilance [11].

Oral calcium supplementation is a practical preventive strategy. A randomized placebo-controlled crossover study found fewer citrate-associated donor symptoms after oral calcium carbonate [12]. Subsequent prospective and observational studies have supported several oral calcium regimens, although formulations, doses, timing, apheresis indications, and co-administered intravenous calcium have varied [13-17]. Evidence on the combined contribution of donor characteristics, procedural citrate exposure, and calcium use in routine plateletpheresis practice in Bangladesh remains limited. The available Bangladeshi evidence has primarily described plateletpheresis-related adverse events and has not jointly evaluated donor characteristics, procedural citrate exposure, and documented calcium use [11]. Consequently, international evidence may not fully reflect local donor profiles, procedural practices, or calcium-administration patterns. We therefore examined donor- and procedure-related factors associated with clinically identified citrate toxicity, with particular attention to body mass index (BMI), pre-donation platelet count, ACD-A volume, and pre-procedure oral calcium. We hypothesized that these factors would be associated with clinically assessed, symptom-based citrate toxicity.

## Materials and methods

Study design and setting

We conducted an analytical observational cross-sectional study in the Department of Transfusion Medicine, Dhaka Medical College Hospital, Dhaka, Bangladesh, between June 1, 2023, and May 31, 2024. The unit of analysis was one plateletpheresis donation encounter; exposure and clinical outcome data were recorded within that encounter. Reporting was prepared with reference to the Strengthening the Reporting of Observational Studies in Epidemiology (STROBE) statement [18].

Participants

Prospective apheresis platelet donors were screened according to national donor-selection guidance. The inclusion and exclusion criteria are presented in Table 1.

**Table 1:** Inclusion and exclusion criteria.

Inclusion criteria	Exclusion criteria
Age 18-60 years	Otherwise ineligible for plateletpheresis
Weight >50 kg	Aspirin or an aspirin-containing product taken within the preceding 48 h
Hemoglobin >12.5 g/dL	Declined to provide written informed consent
Platelet count >150×10⁹/L	-
Free of current illness	-
No clopidogrel or ticlopidine use during the preceding 14 days	-
Negative tests for HIV, hepatitis B, hepatitis C, syphilis, and malaria	-

Candidate factors and exposure classification

A total of 60 eligible donors were enrolled. Candidate donor factors were age, BMI, hematocrit, and pre-donation platelet count. Procedure-related factors were documented as receipt of one 500 mg oral calcium carbonate tablet 15 min before plateletpheresis, number of cycles, procedure duration, and total ACD-A volume. Calcium administration was non-randomized. The available study records documented whether calcium was given but did not specify a standardized clinical criterion or reason for providing calcium to selected donors; therefore, selection bias, residual confounding, and confounding by indication are possible. Post-donation platelet count, platelet yield, and product volume were summarized as same-encounter characteristics but were not treated as baseline donor factors.

Plateletpheresis and monitoring

Plateletpheresis was performed using a Haemonetics MCS+ LN 9000 (Braintree, MA: Haemonetics Corp.) cell separator with a platelet (PLT) and plasma (PLS) set with filter (Ref. 997CF-E), and following standard departmental procedures. Whole blood was withdrawn through venous access, platelets were separated by centrifugation, and the remaining components were returned to the donor. ACD-A was used at a citrate-to-whole-blood ratio of 1:11. The draw and return flow rates were each set at 80 mL/min. The target platelet yield was 3.0-3.5×10^11^ per unit; the processed blood volume ranged from 1,974 to 2,960 mL, and the total ACD-A volume ranged from 270 to 331 mL. A separate citrate infusion rate was not recorded in the extracorporeal circuit. Donors were observed continuously throughout the procedure and until discharge.

A 5 mL venous blood sample was obtained approximately 30 min before plateletpheresis using aseptic precautions. Hematocrit and platelet count were documented before donation; platelet count was reassessed after donation, and platelet yield and final product volume were recorded. This study did not measure total or ionized calcium; consequently, the outcome represents clinical citrate toxicity rather than biochemically confirmed hypocalcemia.

Outcomes

The outcome was the presence of any prespecified clinical feature of citrate toxicity during the donation encounter. Features were circumoral paresthesia, acral paresthesia, nausea, vomiting, muscle cramping, coldness, jaw cramping, forehead tightness, dry mouth, stridor, convulsions, carpopedal spasm, tetany, Trousseau's sign, and Chvostek's sign. Symptoms and signs were recorded through direct observation, donor report, and general and systemic examination. The binary citrate-toxicity variable agreed with a composite of the symptom fields. Individual manifestations were described among affected donors. Donors were assessed during routine continuous monitoring by the clinical staff responsible for the procedure. The source records did not include a standardized adverse-reaction grade, evaluator identifier, symptom-onset time, treatment administered, or a formal differential assessment for anxiety or vasovagal reactions. Total and ionized calcium were not measured.

Statistical analysis

Data analysis was performed using Stata software version 17.0 (College Station, TX: StataCorp LLC). Continuous variables are presented as mean±standard deviation and were compared by citrate-toxicity status using Welch's independent-samples t-test. Categorical variables are presented as n (%) and were compared using two-sided Fisher's exact tests when sparse. Separate modified Poisson models with robust sandwich variance estimated crude prevalence ratios (PRs) for candidate factors [19]. To limit overfitting with 28 outcome events, the clinically selected adjusted model represented the following four distinct domains: calcium exposure (no calcium versus calcium), donor habitus (BMI, per 1 kg/m^2^), baseline platelet status (pre-donation platelet count, per 0.5×10^11^/L), and citrate exposure (ACD-A, per 50 mL). Procedure duration, cycles, and product volume were not entered with ACD-A because they were strongly correlated same-encounter measures (Pearson's r=0.93-1.0). Age and hematocrit are reported descriptively and with crude PRs but were not entered into the parsimonious model. Two-sided p<0.05 was considered statistically significant. Given the 28 outcome events, the adjusted estimates are exploratory and may be unstable; we therefore used a clinically selected parsimonious model and interpret the estimates cautiously.

## Results

Participants and characteristics

All the participants were male; the mean age was 26.65 years, and 28 (46.7%) met the clinical definition of citrate toxicity. Citrate toxicity occurred in 6/30 (20.0%) donors who received pre-procedure calcium and 22/30 (73.3%) donors who did not. Donors with citrate toxicity had lower mean BMI and lower pre- and post-donation platelet counts than donors without toxicity. Their procedures yielded a larger product volume, involved more cycles, lasted longer, and used more ACD-A. Age and hematocrit did not differ materially. Pre-procedure calcium exposure was documented in 6/28 donors with toxicity (21.4%) and 24/32 without toxicity (75.0%; p<0.001) (Table 2).

**Table 2:** Donor and plateletpheresis characteristics by citrate-toxicity status. Values are mean±standard deviation unless stated otherwise. P-values are from Welch's independent-samples t-tests for continuous variables and two-sided Fisher's exact test for calcium exposure.

Characteristic	Citrate toxicity (n=28)	No citrate toxicity (n=32)	p-Value
Age (years)	26.43±5.79	26.84±5.35	0.775
Body mass index (kg/m²)	24.03±2.46	25.56±2.47	0.020
Hematocrit (%)	42.49±1.83	41.88±1.99	0.222
Pre-donation platelets (×10^11^/L)	2.53±0.37	3.23±0.44	<0.001
Post-donation platelets (×10^11^/L)	1.69±0.37	2.38±0.44	<0.001
Platelet yield (×10^11^/unit)	3.11±0.10	3.40±0.18	<0.001
Product volume (mL)	268.71±29.37	225.63±27.18	<0.001
Number of cycles	5.36±0.73	4.28±0.77	<0.001
Procedure duration (min)	65.75±8.25	50.91±5.13	<0.001
ACD-A volume (mL)	333.39±42.05	259.13±27.01	<0.001
Pre-procedure calcium exposure, n (%)	6 (21.4)	24 (75.0)	<0.001

Clinical manifestations of citrate toxicity

Among the 28 donors with citrate toxicity, circumoral paresthesia was the most common manifestation (21/28, 75.0%), followed by dry mouth (12/28, 42.9%), acral paresthesia (9/28, 32.1%), nausea (7/28, 25.0%), and coldness (6/28, 21.4%). Jaw cramping and forehead tightness occurred in four (14.3%) and five (17.9%) affected donors, respectively. Stridor, carpopedal spasm, Trousseau's sign, and Chvostek's sign were each documented once. No convulsion or tetany occurred. Manifestations were not mutually exclusive (Table 3).

**Table 3:** Manifestations among donors with citrate toxicity (n=28). Values are n (%) among the 28 donors classified as having citrate toxicity. Manifestations were not mutually exclusive.

Manifestations	n (%)
Circumoral paresthesia	21 (75.0)
Dry mouth	12 (42.9)
Acral paresthesia	9 (32.1)
Nausea	7 (25.0)
Coldness	6 (21.4)
Forehead tightness	5 (17.9)
Jaw cramping	4 (14.3)
Vomiting	2 (7.1)
Muscle cramping	2 (7.1)
Stridor	1 (3.6)
Carpopedal spasm	1 (3.6)
Trousseau's sign	1 (3.6)
Chvostek's sign	1 (3.6)
Convulsion	0 (0)
Tetany	0 (0)

Factors associated with citrate toxicity

In univariable analyses, absence of pre-procedure calcium, lower BMI, lower pre-donation platelet count, more cycles, longer procedure duration, and greater ACD-A volume were associated with citrate toxicity. In the adjusted model, absence of calcium was associated with 4.42 times the prevalence of toxicity (95% CI: 2.59-7.56), each 1 kg/m^2^ higher BMI was associated with 9% lower prevalence (aPR: 0.91, 95% CI: 0.85-0.98), and each additional 50 mL of ACD-A was associated with 65% higher prevalence (aPR: 1.65, 95% CI: 1.24-2.18). Pre-donation platelet count was no longer statistically significant after adjustment (aPR: 0.79 per 0.5×10^11^/L, 95% CI: 0.56-1.12) (Table 4).

**Table 4:** Crude and adjusted prevalence ratios for factors associated with citrate toxicity. PRs and 95% CIs are from a modified Poisson regression with robust sandwich variance. The adjusted model included calcium exposure, BMI, pre-donation platelet count, and ACD-A volume. Dashes indicate variables not entered into the parsimonious adjusted model. ACD-A: anticoagulant citrate dextrose solution A; aPR: adjusted prevalence ratio; PR: prevalence ratio

Factors (comparison)	Crude PR (95% CI)	p-Value	Adjusted PR (95% CI)	p-Value
No calcium versus calcium	3.67 (1.74-7.75)	<0.001	4.42 (2.59-7.56)	<0.001
Age, per 5 years higher	0.96 (0.74-1.26)	0.782	-	-
BMI, per 1 kg/m² higher	0.88 (0.80-0.98)	0.014	0.91 (0.85-0.98)	0.011
Hematocrit, per 1% higher	1.09 (0.95-1.25)	0.202	-	-
Pre-donation platelets, per 0.5×10^11^/L higher	0.48 (0.38-0.60)	<0.001	0.79 (0.56-1.12)	0.192
ACD-A, per 50 mL higher	1.97 (1.58-2.45)	<0.001	1.65 (1.24-2.18)	<0.001
Cycles, per one higher	1.98 (1.53-2.57)	<0.001	-	-
Duration, per 10 min higher	2.01 (1.61-2.51)	<0.001	-	-

## Discussion

In this single-center observational cross-sectional analysis, citrate toxicity was clinically identified in 46.7% of plateletpheresis donors. Three factors remained associated in the parsimonious adjusted model as follows: absence of pre-procedure oral calcium, lower BMI, and greater ACD-A volume. Pre-donation platelet count showed a strong crude association but attenuated after adjustment for calcium exposure, BMI, and citrate volume. Age and hematocrit were not associated in univariable analyses.

The associations with lower BMI and greater citrate exposure are biologically plausible. Donors with smaller body size may receive a larger effective citrate dose relative to distribution volume, while greater citrate delivery increases ionized-calcium chelation. Previous apheresis studies have linked lower body size, greater processed volume, longer procedures, and higher citrate exposure with donor reactions [4,8-10]. In this study, ACD-A volume, procedure duration, cycles, and product volume were highly correlated and should be viewed as overlapping markers of procedural intensity rather than separate independent effects.

Absence of calcium exposure was associated with substantially higher toxicity prevalence after adjustment. The direction agrees with a randomized placebo-controlled crossover study of oral calcium carbonate [12], a prospective plateletpheresis study [13], and reports of other oral or intravenous calcium regimens [14-17]. However, because calcium administration was non-randomized and its clinical indication was not standardized in the available record, residual confounding, including confounding by indication, may explain part or all of this association. It should not be interpreted as evidence that calcium prophylaxis prevents citrate toxicity.

The attenuation of the pre-donation platelet count association after including ACD-A suggests that baseline platelet count and procedural citrate exposure capture related pathways. Lower starting platelet counts can require longer or more intensive collection to reach a target yield, which may increase citrate delivery. Because total ACD-A, duration, and cycles were recorded over the same encounter in which symptoms developed, the analysis cannot determine how much exposure occurred before symptom onset or establish temporal direction.

Strengths of this study include complete outcome and model data for all 60 donors, a prespecified clinical symptom set, and consideration of donor, prophylactic, and procedural domains. Reporting adjusted PRs also provides an interpretable association measure for a common outcome.

This study has some important limitations. Its cross-sectional observational framing cannot establish causality, and temporality is especially uncertain for total ACD-A volume, duration, and cycles. The small single-center sample included only 60 male donors and 28 outcomes, so the multivariable model was deliberately limited and requires external validation. The composite endpoint included mild, subjective, and non-specific features. Total and ionized calcium, magnesium, citrate infusion rate, reaction severity, electrocardiographic changes, calcium-related adverse effects, and donor return behavior were not measured. Symptom onset timing relative to citrate dose and rescue protocols were unavailable. Residual confounding and multiple exploratory comparisons remain possible. Calcium administration was non-randomized, and a standardized indication for its use was not recorded; calcium recipients may therefore have differed systematically from non-recipients. The absence of standardized reaction grading, symptom timing, treatment records, and formal differentiation from anxiety or vasovagal reactions may have caused outcome misclassification. These limitations, together with the limited number of outcome events, mean that the adjusted estimates may be unstable and should be considered hypothesis-generating.

The findings support closer monitoring of donors with lower BMI and procedures requiring greater citrate exposure, together with consistent documentation of calcium use and symptom onset. They do not by themselves justify a causal prevention protocol. Larger prospective multicenter studies should record citrate infusion rate and cumulative dose at symptom onset, use standardized reaction grading, measure serial ionized calcium, and evaluate calcium prophylaxis with an appropriately controlled design.

## Conclusions

Among plateletpheresis donors, clinical citrate toxicity was associated with the absence of pre-procedure oral calcium, lower BMI, and greater ACD-A volume. Lower pre-donation platelet count was associated in univariable analysis but not after adjustment. These findings are exploratory and hypothesis-generating. In particular, the observed association with calcium cannot be interpreted as evidence of prophylactic efficacy because allocation was non-randomized and potentially confounded. These factors may help identify donors and procedures requiring closer surveillance; however, the small, all-male sample, same-encounter measurements, symptom-based outcome, absence of ionized calcium data, and possible model instability preclude causal or predictive claims.
